# Is Medication-Related Osteonecrosis of the Jaws (MRONJ) Associated to Cyclin-Dependent Kinase (CDK) 4/6 Inhibitors? A Word of Cautiousness. Comment on Marcianò et al. Medication-Related Osteonecrosis of the Jaws and CDK4/6 Inhibitors: A Recent Association. *Int. J. Environ. Res. Public Health* 2020, *17*, 9509

**DOI:** 10.3390/ijerph181910143

**Published:** 2021-09-27

**Authors:** Vittorio Fusco, Manuela Alessio, Pamela Francesca Guglielmini, Maura Vincenti, Antonella Fasciolo, Maura Rossi

**Affiliations:** 1 Oncology Unit and Centro Documentazione Osteonecrosi, Azienda Ospedaliera “SS Antonio e Biagio e Cesare Arrigo”, 15121 Alessandria, Italy; pfguglielmini@ospedale.al.it (P.F.G.); mvincenti@ospedale.al.it (M.V.); mrossi@ospedale.al.it (M.R.); 2 Study CoordinatorOncology Unit, Ospedale “Michele e Pietro Ferrero”, 12060 Verduno (CN), Italy; malessio@aslcn2.it; 3 Maxillofacial Surgery Unit, Azienda Ospedaliera “SS Antonio e Biagio e Cesare Arrigo”, 15121 Alessandria, Italy; afasciolo@ospedale.al.it

Marcianò et al. launched an alert in this journal about a possible association between medication-related osteonecrosis of the jaws (MRONJ) and cyclin-dependent kinase (CDK) 4/6 inhibitors in breast cancer patients [1].

The report is based on the observation of six cases of MRONJ in breast cancer patients treated with zoledronic acid and/or denosumab (as antiresorptive therapy for bone metastases) together with palbociclib (5) or abemaciclib (1) (as antitumor treatment), among a total of 16 MRONJ cases observed in an oral care referral center, between the first quarter of 2018 and the first quarter 2020.

In appearance this observation may appear similar to what was observed in patients receiving a long list of anticancer drugs (sunitinib, bevacizumab, aflibercept, sorafenib, cabozantinib, everolimus, temsirolimus, etc) which were demonstrated to induce MRONJ or to increase the risk of MRONJ [2,3,4,5,6,7].

Inhibitors of CDK 4/6, often simply named CDK inhibitors or cyclin inhibitors, include palbociclib, ribociclib, abemaciclib, and other drugs now under investigation. They act on the CDK-RB1-E2F pathway which is disrupted in cancer cells, and they are prescribed alone or, more often, together with drugs which prevent the downstream estrogen-dependent stimulation of cancer cells in most breast cancers [8]. They have been tested and approved for the treatment of estrogen-dependent HER2-negative metastatic breast cancer patients [9,10,11].

Consequently, CDK inhibitors have become a largely adopted treatment option in endocrine dependent metastatic breast cancer. As skeletal is one of the main metastatic sites of this tumor type, it not surprising that a large number of patients treated with CDK inhibitors (those with bone metastases) will also receive one of the available antiresorptive agents (also known as Bone Modifying Agents, BMAs, or Bone Targeting Agents, BTAs): bisphosphonates (pamidronate, ibandronate, and zoledronic acid) or denosumab (a RANKL inhibitor), which are drugs more frequently associated to MRONJ.

Why have drugs different from antiresorptive agents (i.e., sunitinib, bevacizumab, etc.) been associated with MRONJ by researchers?

1. MRONJ cases were observed among patients receiving these drugs with no concomitant antiresorptive agent treatment; this constitutes a real proof of evidence [2];
2. A higher risk for MRONJ has been registered in patients receiving both antiresorptive and antiangiogenic agents [4,12,13,14,15];
3. A plausible mechanism of action is known (i.e., antiangiogenic activity in the case of bevacizumab, aflibercept, but also sunitinib and other tyrosine-kinase inhibitors) [2,3].

The question is whether CDK inhibitors answer to the aforementioned criteria, suggesting a causal association to MRONJ.

1. To the best of our knowledge, there were no reported or published cases of MRONJ among patients receiving CDK inhibitors without antiresorptive agents in some thousand patients treated with palbociclib, ribociclib, and abemaciclib, as reported in randomized trials and other drug safety reports (i.e., those by Food and Drug Administration) [16,17,18];
2. A higher rate of MRONJ should have been registered in patients receiving both CDK inhibitors and antiresorptive agents (bisphosphonates and/or denosumab) in comparison with patients treated with antiresorptive agents alone. This kind of data might be obtained by randomized trials comparing CDK inhibitors and placebo (with hormone therapy), and has not yet been reported to the best of our knowledge. However, it could even be registered in real life data, evaluating MRONJ rate in large patient populations with an adequately long follow up and comparing it with that of similar patient population.
3. In the literature on CDK inhibitors, we found no apparent mechanism for induction of MRONJ.

Finally, even if limited, we wish to report data from our hospital oncology unit as an example of what can expected from observation in a practice clinical setting. In the last 3 years, 24 bone metastatic breast cancer patients received CDK inhibitors together with antiresorptive drugs: 12 with palbociclib, 11 with ribociclib, and 1 with abemaciclib; 12 received denosumab and 12 zoledronic acid. Two patients (2/24, 8.3%) showed MRONJ. The first woman had received both palbociclib and denosumab for 30 months at the ONJ onset. Another woman had received a sequence of intravenous zoledronic acid, oral ibandronate, and subcutaneous denosumab for 36 months at the ONJ diagnosis, and has received palbociclib for five months only (stopped due to heavy symptomatic skeletal progression) one year before. Furthermore, no cases of MRONJ were registered among the other 40patients receiving CDK inhibitors (20 palbociclib, 19 ribociclib, 1 abemaciclib) without antiresorptive agents.

In conclusion, in our opinion, at this moment, an association between CDK 4/6 inhibitors and MRONJ is not inferable.

## Data Availability

No new data were created or analyzed in this study. Data sharing is not applicable to this article.
